# The relationship between socioeconomic indices and potentially zoonotic pathogens carried by wild Norway rats: a survey in Rhône, France (2010–2012)

**DOI:** 10.1017/S0950268814001137

**Published:** 2014-05-16

**Authors:** F. AYRAL, J. ARTOIS, A.-L. ZILBER, F. WIDÉN, K.C. POUNDER, D. AUBERT, D. J. BICOUT, M. ARTOIS

**Affiliations:** 1 WildTech, PERS, Université de Lyon-VetAgro Sup, Marcy l'Etoile, France; 2 Université de Rennes 2, Rennes Cedex, France; 3 The National Veterinary Institute (SVA), Department of Virology, Immunobiology and Parasitology, Uppsala, Sweden; 4 Institute of Integrative Biology, University of Liverpool, Liverpool, UK; 5 Laboratory of Parasitology, EA 3800, SFR CAP-Santé FED 4231, National Reference Centre on Toxoplasmosis, University of Reims Champagne-Ardenne, Reims Cédex, France; 6 Biomatématiques et Epidémiologie, EPSP-TIMC, UMR CNRS 5525, UFJ, VetAgro Sup, Marcy l'Etoile

**Keywords:** Hepatitis E virus, *Leptospira interrogans*, Norway rats, *Toxoplasma gondii*, Seoul virus

## Abstract

*Leptospira interrogans*, hantaviruses (particularly Seoul virus), hepatitis E virus (HEV), and *Toxoplasma gondii* are rat-associated zoonoses that are responsible for human morbidity and mortality worldwide. This study aimed to describe the infection patterns of these four pathogens in wild rats (*Rattus norvegicus*) across socioeconomic levels in neighbourhoods in Lyon, France. The infection or exposure status was determined using polymerase chain reaction or serology for 178 wild rats captured in 23 locations; additionally, confirmatory culture or mouse inoculation was performed. Multivariate logistic regression analyses were used to investigate whether morphological and socioeconomic data could predict the infection status of the rats. This study revealed that the rat colony's age structure may influence the prevalence of *L. interrogans*, hantavirus, and HEV. In addition, areas with high human population densities and low incomes may be associated with a greater number of infected rats and an increased risk of disease transmission.

## INTRODUCTION

Wildlife is estimated to be responsible for 72% of emerging infectious diseases in humans [1]. Key among wildlife species, *Rattus norvegicus* is prevalent worldwide and harbours rat-borne zoonoses (RBZs) [2], such as *Leptospira interrogans*, hantaviruses, hepatitis E virus (HEV), and *Toxoplasma gondii*. These RBZs are responsible for potentially life-threatening human infections and have raised public health concerns in Europe.

Leptospirosis (caused by *L. interrogans sensu lato*), an acute bacterial infection in humans, was recently recognized as an emerging public health problem [3]. *Leptospira* are maintained by a wide range of hosts, and *R. norvegicus* is reportedly the primary carrier [4]. The bacteria are shed in the hosts’ urine and can survive for prolonged periods. Occupational and recreational exposures to contaminated water bodies in proximity to various host species are commonly reported risk factors. However, cities, particularly environments with poor hygiene, are the primary source of rat-associated leptospirosis [5].

Haemorrhagic fever with renal syndrome is a mild to severe human disease caused by viruses from the genus *Hantavirus*, family Bunyaviridae [6]. Each hantavirus species is carried by a specific rodent host species; Seoul virus (SEOV) has been found to be carried by *R. norvegicus* worldwide [2]. Unlike in Asia, few confirmed occurrences were reported in Europe before the recent emergence in the UK and France [7, 8]. Hantavirus is shed in the excreta of infected hosts, and the primary transmission pathways are the inhalation of suspensions of contaminated dust and the passage of infected saliva through bites [9]. Areas with growing rodent populations are at increased risk for human infection [10].

HEV is widespread and causes acute hepatitis in humans. A variety of HEV strains have been identified in Norway rats from Europe without any evidence of zoonotic pathogenicity [11, 12]. However, zoonotic HEV has been reported in the USA [13], confusing the role of rats as a disease reservoir for humans and livestock.

Toxoplasmosis is a highly prevalent parasitic disease worldwide and is caused by the protozoan *T. gondii*. Humans become infected after ingesting food contaminated with *T. gondii* oocysts. Vertical transmission is particularly concerning for humans, as *in utero* infection can cause abortion or congenital defects [14]. Although cats are the only hosts known to excrete the infectious form, the parasite cycle involves most warm-blooded vertebrate species, including Norway rats. As felids’ prey, rats are important in the parasite life cycle and in human toxoplasmosis epidemiology.

The Norway rat is a synanthropic species that uses human habitats for shelter and food. Impoverished inner cities have been targeted for RBZ investigations because it is assumed that poor hygiene conditions, infrastructure disrepair, and low health status may lead to contact between rats and humans, and potentially to RBZ transmission [15]. Conditions in impoverished cities may also be related to high numbers of infected and shedder rats, which are responsible for rapidly spreading pathogens in human habitats and increased human exposure. However, no previous studies have investigated whether impoverished inner cities have a higher prevalence of infected rats. A description of RBZ carriage in Norway rats across socioeconomic indices increases the knowledge of the related health risks. A more complete understanding of zoonotic infection in rats would mitigate their role in spreading zoonotic diseases by increasing awareness among healthcare professionals and supporting the development of appropriate surveillance.

The aims of the present study were (1) to describe the prevalence of the four pathogens in the Lyon region, France, (2) to identify the risk factors for infection in Norway rats, and (3) to define the potential socioeconomic indicators for human exposure in the Lyon region. Thus, trapping was performed on an urbanization gradient in and around Lyon. Multivariate models were built to predict the pathogen carriage status of the rats; morphological and socioeconomic data were used as independent variables.

## METHODS

### Study area

The study area comprised 23 locations in the Rhône department (i.e. administrative spatial unit) of France, which encompasses the city of Lyon and 14 farms within 30 km of our laboratory (45° 47′ 30·93′ N; 4° 42′ 30·28′ E). This area contains a range of urbanization levels. Farms were requested to participate based on their inclusion in previous surveys, and all agreed. The dwellings and public areas were selected based on high rat concentrations reported by the Hygiene Service of Lyon.

Most farms were medium to large size (i.e. > 100 animals) with mixed livestock. The nine urban trapping locations were distributed in dwelling areas, public gardens, waste disposal areas, and an industrial area in the city centre and suburbs.

### Sampling

The survey was subdivided into 6-month periods: (1) September 2010 to February 2011 and (2) October 2011 to March 2012. To account for possible seasonal variation in the pathogen prevalence, the two periods encompassed the same season. The sampling size (*n*≈90) was calculated using a 95% confidence level, a relative accuracy of 50%, and an expected prevalence of 10–30% [16]. To account for possible variation in the pathogen prevalence among different urbanization levels, the same sample size was used in areas of low and high population density. During the two periods, 178 free-living Norway rats were trapped in areas of low (*n* = 94) and high (*n* = 84) population density.

Most rats were captured with small (28 cm × 9 cm × 9 cm) or large (50 cm × 15 cm × 15 cm) single-catch rat traps. The captured rats were transported to the laboratory and immediately anaesthetized using isoflurane. A blood sample was obtained by cardiac puncture. Subsequently, the rats were sacrificed by cervical dislocation. The rats that succumbed to capture were frozen at −20°C and thawed on the day of dissection. Data were recorded on weight, size, sex, sexual maturity (i.e. the presence of seminal vesicles in males and a developed uterus in females) and pregnancy. Lung and liver lobe fragments weighing between 50 and 100 mg were collected, and the kidney was removed. The heart was incorporated in an antibiotic solution with 1·2 × 10^−5^ g/ml amoxicillin (Amoxicilline Panpharma, France), 120 UI/ml penicillin and 120 *μ*g/ml streptomycin (Pen-Strep Liq 10 000, Gibco, USA). Faecal samples were collected from the rectum, and all tissue and faecal samples were stored at −80°C immediately following collection.

The authors assert that all procedures contributing to this work comply with the ethical standards of the relevant national and institutional guides on the care and use of laboratory animals (agreement no. 69-020931).

### Screening protocols

*L. interrogans* colonization of the kidney was assessed via culture [17] and a pathogen-specific *L. interrogans* TaqMan real-time polymerase chain reaction (PCR) kit (TaqVet PathoLept kit, LSI, France); additionally, the *rpoB* gene was targeted. Lung tissues were screened for hantavirus using a nested pan-hantavirus reverse transcription (RT)–PCR selective for partial polymerase large segment (L) gene sequences, subsequent molecular typing was performed to determine the hantavirus species as previously described [18]. The HEV viral load in the liver and faeces was analysed using TaqMan real-time PCR reagents specific to rat-strain HEV and genotypes 1–4 [19]. Sera extracted from blood-soaked blotters were tested for *T. gondii* antibodies using a modified agglutination test (MAT) that detects *T. gondii*-specific IgG antibodies [20]. The hearts of animals with a positive agglutination (starting dilution 1:6) were bioassayed in mice to isolate viable *T. gondii* [21].

### Prevalence calculation

*L. interrogans*, SEOV, and HEV were screened based detecting pathogen nucleic acid sequences or the microorganism itself; rats testing positive were considered to be infected and potential shedders of the pathogens. The rats with MAT-positive results were considered to be exposed because the presence of antibodies against the pathogen could result from previous or current contact with the pathogen. The prevalence of infected or exposed rats was defined as the proportion of rats with any positive test results. The apparent prevalence was restricted to the population sampled since and the administrative unit of the Rhône department

### Variables

Two categories of explanatory covariates were considered: rat-related and socioeconomic covariates. Rat survival during the trapping was also considered, as death and freeze–thaw cycles before dissection could alter the sample quality and underestimate the prevalence.

The rat data included the species (determined by external morphology and macroscopic observations), sex, weight, size, and approximate age, based on sexual maturity and pregnancy. A body mass metric was constructed from the residuals that resulted from the regression of weight on size [22]. The capture success, a proxy of the population size, was accurately defined in five sites by

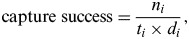
where *n*_*i*_ is the number of rats captured, *t*_*i*_ the number of traps set and *d*_*i*_ the number of days of trapping at the site *i*.

The extent and features of urbanization were characterized using the socioeconomic data. Eleven candidate variables were extracted from the 2009 national census data provided by the Institut National de la Statistique et des Etudes Economiques. Remotely sensed data were obtained at the smallest geographical unit, which is the Ilot Regroupé pour l'Information Statistique (IRIS). Each IRIS contains 2000 inhabitants. The socioeconomic variables are displayed in Table 1.

**Table 1.** Description of socioeconomic covariates

Covariates	Name of covariate	Definition
Human population density	Human population	Population/surface (km)^2^
Median income of the population	Income	Median incomes of the population included in the IRIS
Population growth from 1999 to 2009	Population growth	Population in 2009/population in 1999
Percentage of 20–64 years age group	Population age	20–64 years age group/total population
Percentage of flats	Per cent flat	Number of flats/number of flats + houses
Percentage of small flats	Per cent small flat	Number of 1- and 2-room dwellings/number of dwellings
Ratio of renters and owners	Ratio renter/owner	Number of renters/number of owner of their residency
Ratio of low-income dwellings (LID)	ratio LID	Number of LID rented/number of other rented dwellings
Percentage of ungraduated population	Level of graduation	Population aged >15 years without a diploma/population aged >15 years
Ratio of large and small families	Family size	Family with at least three children/family with two or fewer children
Ratio of unemployment	Rate of unemployment	Number of unemployed people/number of employed people

### Spatial analysis

The location and number of rats in each trapping area that tested positive and negative were mapped using ArcGIS version 9.3 (ESRI, USA). Spatial clusters of high and low pathogen prevalence were identified using the Getis-Ord Gi* statistic. The local sum for features and the feature's neighbours were compared proportionally to the sum of all features. When the local sum was statistically significantly different from the expectation, the region was denoted as having low or high prevalence.

To identify the potential socioeconomic proxies of RBZ, the candidate variables from the census data sets were extracted from the IRIS units with trapping locations. An approach using various boundaries for the quadrats when extracting data was implemented to verify the effects of different spatial scales on the risk factors [23]. The socioeconomic data were extracted using four quadrat sizes: 100 m × 100 m, 200 m × 200 m, 400 m × 400 m, and 800 m × 800 m. The socioeconomic metrics for each quadrat were calculated by weighting the remotely sensed data by the percentage of the IRIS surface that was included in the quadrat. For each spatial level of analysis, a new data set was obtained by spatial query using the software R project version 3.0.1 (R Development Core Team, Austria) and ArcGIS version 9.3 (ESRI, Redland, CA, USA).

### Statistical analysis

To identify the characteristics associated with each pathogen's carriage status, distributions of the explanatory variables were examined within the samples testing positive and negative. *χ*^2^ tests and Wilcoxon tests were used, as appropriate, and an alpha level of 0·05 was used to reject the null hypothesis. For each pathogen, the potential effect of the population size on the number of infected rats in the trapping sites was assessed by testing the Pearson correlation between the capture success and the related number of infected rats. An *α*-level of 0·05 was used to reject the absence of correlation. The result obtained determined whether the capture success had to be considered in the models.

Statistical modelling was performed to evaluate the use of rat-related criteria and socioeconomic indices as predictors of exposure or infection in rats. All remotely sensed data were transformed to be normally distributed when they were incorporated into the models, and missing data were not considered. Simple logistic regression (SLR) was used to examine the relationship between each pathogen's prevalence and the explanatory variables. Covariates that were significantly associated with pathogen infection or exposure at an *α*-level of 0·10 were considered for further analysis. To select independent variables, rat-related covariates were tested for multicollinearity using Spearman's rank correlation (*ρ* > 0·7) with the Hmisc package in R, whereas socioeconomic covariates were described with a principal component analysis (PCA) using the FactoMineR package in R. From the PCA result, one variable per main axis was chosen based on the comprehensiveness of the data. Selected covariates were incorporated into multivariate models. The variation inflation factors were calculated with the car package in R to verify a low effect of potential collinearity (<5).

When the effect of rat death during capture was significantly associated with the infection status in SLRs, the data set was adjusted to account for a possible sample alteration effect.

For each pathogen, two multivariate logistic regression models were implemented. The selected covariates were incorporated into a generalized linear model (GLM) as fixed effects. The final GLM was selected using Aikake's Information Criterion (AIC) to balance model fit and parsimony. Then, the adjusted effects of the variables included in the final GLM were estimated with a generalized linear mixed model (GLMM) to control for the random effect of trapping locations and to account for the potential effects of clustering.

The remotely sensed data extracted from the quadrats were considered for each socioeconomic variable included in the final GLMs. A new set of models was created to account for the possible influence of changing spatial scales on the adjusted effects.

## RESULTS

### Rat population

During the two collection periods (37 and 39 days), 178 rats were trapped and sampled (54% male). About two-thirds (63%) of the rats were adults, and 18 of the 58 adult females were pregnant (31%). The ratio of sexually immature to mature rats was similar between the farm (ratio = 40:54) and city centre (ratio = 43:58) sites. However, at the city centre sites, the ratio was lower in dwellings (ratio = 5:37) than in industrial areas (ratio = 19:4).

### Pathogen detection

Norway rats were the primary carrier and potential shedder of *L. interrogans* (26% prevalence), SEOV (14%), and rat-specific-HEV (14%). Eight per cent of the rats with a MAT titre of ⩾1:6 were previously exposed to *T. gondii*. Among the 77 hearts, 58 were bioassayed in mice. Twenty-four bioassays were inconclusive because the mice did not survive past 48 h post-infection, and *T. gondii* was not isolated from the 34 other bioassays (Table 2).

**Table 2.** The percentage of positive tests for each pathogen and the distribution of positive test results among the performed tests

Pathogens (no. of rats tested)	*L. interrogans* (*n* = 171)		SEOV (*n* = 127)	HEV (*n* = 142)		*T. gondii* (*n* = 77)	
Percentage of positive tests	26% (*n* = 45/171)		14% (*n* = 18/127)	14% (*n* = 20/142)		8% (*n* = 6/77)	
95% Confidence intervals	20–33%		8–20%	8–20%		2–14%	
Tests	RT–PCR	Culture	Nested PCR	RT–PCR	RT–PCR	MAT	Bioassay
Materials	Kidney	Kidney	Lung	Liver	Feces	Blood blot	Heart
No. of positive tests	44	22	18	18	12	6	0

Among 120 Norway rats screened for *L. interrogans*, SEOV, and HEV, 50 (42%) were infected with at least one pathogen. A single pathogen was detected in the majority of infected rats (*n* = 31/50); the rest were co-infected with at least two agents. The proportion of rats infected with SEOV or HEV (*n* = 34) and co-infected with *L. interrogans* (*n* = 19) was significantly higher than the proportion of rats infected by *L. interrogans* alone (Pearson‘s *χ*^2^ = 14·63, d.f. = 1, *P* = 0·0001); the odds ratio (OR) of being co-infected by *L. interrogans* and SEOV or HEV was 5·54 [95% confidence interval (CI) 2·1–14·4]. Three of the infected rats were simultaneously carrying *L. interrogans*, SEOV, and HEV (see Supplementary online material).

**Fig. 1.**
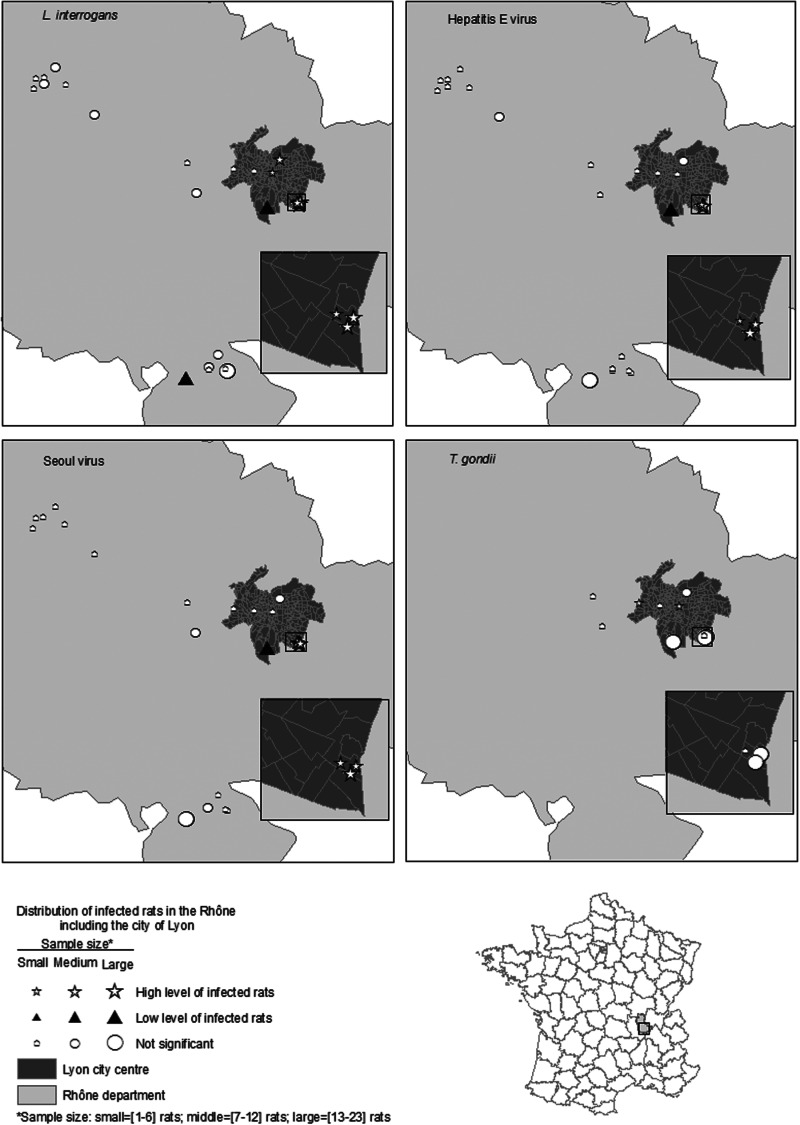
The distribution of clusters of low (full triangle) and high (star) prevalences of the four pathogens detected in rats trapped in the Rhône department, France.

The percentage of infected rats at the trapping sites varied: 0–65% for *L. interrogans*, 0–30% for SEOV, 0–61% for HEV, and 0–25% for *T. gondii*. The spatial distribution of sites with significantly higher or lower infected rats differed among the four pathogens. Nevertheless, high prevalence clusters were encountered for each pathogen in the Lyon region (Fig. 1).

For each pathogen, Pearson's coefficient which characterized the relationship between the capture success and the number of infected rats was not significantly different from zero (all *P* values > 0·14). Thus, no correlation between the capture success and the number of infected individuals could be demonstrated.

### Characterization of infection correlates in rats

#### *L. interrogans* carriage

From the SLR models, the OR of testing positive for *L. interrogans* was greater in sexually mature rats than in sexually immature rats. However, no significant relationship was observed between the infected rats and gender, body condition, or pregnancy (Table 3). The relationship between 10 normalized socioeconomic covariates and *L. interrogans* positivity was statistically significant (all *P* values ⩽0·05).

**Table 3.** Unadjusted and adjusted odds ratios for testing positive for *Leptospira interrogans*, SEOV, HEV or *Toxoplasma gondii* among Norway rats

Covariates	Modes	*L. interrogans status* (*n* = 171)			SEOV status (*n* = 127)			HEV status (*n* = 142)			*T gondii status* (*n* = 77)		
		SLR	GLM	GLMM*	SLR	GLM	GLMM†	SLR	GLM	GLMM‡	SLR	GLM	GLMM
		OR	aOR	aOR	OR	aOR	aOR	OR	aOR	aOR	OR	aOR	aOR
Survival after trapping	Dead	Ref.			Ref.			Ref.			n.a.		
	Alive	**2 (1·1–3·6)**	—	—	2·2 (0·9–5·2)	—	—	1·2 (0·5–2·8)	—	—	—	—	—
Rat preservatives	Frozen	Ref.			Ref			Ref.			n.a.		
	Fresh	**3·3 (1·5–7·4)**	—	—	1·7 (0·6–4·5)	—	—	1·2 (0·5–3·1)	—	—	—	—	—
Sex	Female	Ref.			Ref			Ref.			n.a.		
	Male	1·7 (0·9–3·2)	—	—	1·5 (0·6–3·6)	—	—	1 (0·4–2·4)	—	—	—	—	—
Maturity	Juvenile	Ref.	Ref.		Ref.			Ref.			n.a.		
	Adult	**7·3 (3·1–16·6)**	**4·0 (1·6–9·9)**	**4·0 (1·3–11·9)**	**9 (1·6–55)**	6·2 (1·1–34)	10·5 (0·8–138)	**4·4 (1·5–14·3)**	2·2 (0·7–7)	2·6 (0·5–14·7)	—	—	—
Pregnancy	No	Ref.			Ref.			Ref.			n.a.		
	Yes	1·5 (0·5–4·1)			0·4 (0·1–2·5)	—	—	1·1 (0·2–5)	—	—	—	—	—
Body condition		1 (0·9–1·0)	—	—	1 (0·9–1)	—	—	0·9 (0·9–1·0)	—	—	**1·02 (1·01–1·03)**	—	—
Human population		**3·2 (2·3–4·3)**	**1·8 (1·2–2·7)**	**1·8 (1·1–2·9)**	**1·9 (1·3–2·7)**	—	—	**1·7 (1·2–2·4)**	—	—	1·2 (0·7–2·3)	—	—
Incomes (median)		**0·3 (0·2–0·4)**	**0·6 (0·4–0·9)**	**0·6 (0·3–0·9)**	**0·4 (0·3–0·7)**	**0·5 (0·3–0·8)**	0·4 (0·1–2·1)	**0·3 (0·2–0·5)**	**0·4 (0·2–0·6)**	**0·3 (0·1–0·8)**	1·3 (0·8–2·2)	—	—
Population expansion		1·1 (0·8–1·5)	—	—	1·4 (0·9–2·3)	—	—	1·4 (0·9–2·2)	—	—	1·7 (0·8–3·9)	—	—
Population age		**0·6 (0·4–0·9)**	—	—	0·2 (0·02–1·3)	—	—	0·1 (0·01–1·2)	—	—	0·7 (0·4–1·4)	—	—
Per cent flat		**2·6 (1·9–3·6)**	—	—	1·6 (0·9–2·5)	—	—	1·5 (0·9–2·2)	—	—	0·7 (0·3–1·6)	—	—
Per cent small flat		**3·0 (2·1–4·2)**	—	—	**1·6 (1·1–2·6)**	—	—	**1·5 (1·0–2·3)**	—	—	1·1 (0·4–2·8)	—	—
Ratio renter/owner		**4·2 (2·2–8·0)**	—	—	0·6 (0·3–1·1)	—	—	0·5 (0·3–1·1)	—	—	1·2 (0·8–2·0)	—	—
Ratio LID		**3·1 (2·3–4·3)**	—	—	**2·7 (1·7–4·2)**	—	—	**2·4 (1·6–3·6)**	—	—	0·9 (0·5–1·6)	—	—
Level of graduation		**2·8 (2·1–3·8)**	—	—	**2·2 (1·5–3·2)**	—	—	**2·8 (1·8–4·1)**	—	—	0·1 (0·0–7·3)	—	—
Family size		**3·1 (2·3–3·4)**	—	—	**2·6 (1·7–4·1)**	—	—	**2·6 (1·7–3·9)**	—	—	0·8 (0·5–1·5)	—	—
Rate of unemployment		**3·1 (2·3–4·2)**	—	—	**2·3 (1·5–3·4)**	—	—	**2·5 (1·7–3·8)**	—	—	0·8 (0·5–1·4)	—	—

SLR, Simple logistic regression; GLM, generalized linear model; GLMM, generalized linear mixed model; OR, odds ratio; aOR, adjusted odds ratio; n.a., not applicable; LID, low-income dwelling.

* Variance of the random effect = 0·007.

† Variance of the random effect = 1·7.

‡ Variance of the random effect = 8·9.

All results in bold were significantly different from 1 at *α*⩽0·05.

The multivariate analyses incorporated sexual maturity, human population density (HPD), and median income as independent covariates (multicollinearity test results are not shown). In the final GLM, the OR of being *L. interrogans*-positive was greater in adults than juveniles (OR 4·0, 95% CI 1·6–9·9), and the OR increased with increasing normalized HPD (OR 1·8, 95% CI 1·2–2·7) and decreasing normalized income (OR 0·6, 95% CI 0·4–0·9). The relationship between these variables and *L. interrogans* positivity aligned but with decreased magnitude compared to the SLR. After controlling for clustering trapping locations using GLMMs, the ORs were nearly identical to the ORs obtained with a GLM. The estimated variance for the random effect of trapping locations was 0·007% of the total residual variance. The GLM was maintained for further analysis and used as the simplest model.

Among 155 rats analysed, 30% (*n* = 47) died after trapping and were frozen and thawed before dissection. The OR of being *L. interrogans*-positive was greater for the surviving rats and the fresh samples compared to the frozen rats. Because there was a strong correlation between rat freezing and rat survival in the field, the effects of freezing and survival could not be differentiated. After adjusting the model for rat survival, the relationships between explanatory variables and *L. interrogans* positivity were similar to the results from the model that was not adjusted for survival. The relationships from the adjusted model were as follows: sexual maturity (OR 3·4, 95% CI 1·3–8·5), normalized HPD (OR 1·9, 95% CI 1·2–2·8), and normalized income (OR 0·6, 95% CI 0·4–0·8).

#### SEOV carriage

The results from the SLR models suggested that the OR of being SEOV-positive was greater in sexually mature rats than in sexually immature rats. There was no significant difference in the relationships between body condition, gender or pregnancy, and SEOV positivity. The relationships between seven normalized socioeconomic covariates and SEOV positivity were statistically significant (all *P* values ⩽0·05).

The multivariate analyses incorporated sexual maturity, HPD, and income as independent covariates. In the final GLM, sexual maturity and the normalized incomes of the population were retained. The OR of being SEOV-positive increased with decreasing normalized income (OR 0·5, 95% CI 0·3–0·8) and appeared to be greater in adults than juveniles, although not statistically significant (OR 6·2, 95% CI 1·1–34) for the latter group. In bivariate analyses, the relationship between these variables and SEOV positivity aligned but with increased magnitude. Similar results were obtained by accounting for clustering of the trapping locations in a GLMM. In the selected GLMM, the estimated variance for the random effect of trapping locations was 8·88, accounting for 99% of the total residual variance.

#### HEV carriage

The results from the SLR models suggested that the OR of being HEV-positive was greater in sexually mature rats than sexually immature rats. The relationships between eight normalized socioeconomic covariates and HEV positivity were statistically significant (all *P* values ⩽0·05).

The multivariate analyses used sexual maturity, HPD, and income as independent covariates. In the final GLM, sexual maturity and normalized income were retained. The OR of being HEV-positive increased with decreasing normalized income (OR 0·4, 95% CI 0·2–0·6) and appeared to be greater in adults than juveniles, although results in the latter group were not statistically significant (OR 2·2, 95% CI 0·7–7). In bivariate analyses, the relationship between these variables and HEV positivity aligned but with increased magnitude. Similar results were obtained by accounting for clustering trapping locations using a GLMM. In the selected GLMM, the estimated variance for the random effect of trapping locations was 1·631, accounting for 7% of the total residual variance.

#### *T. gondii* exposure

The results from the SLR models suggested that only body mass was significantly higher in rats exposed to *T. gondii* (*P*⩽0·05). Thus, the multivariate analysis was not investigated.

### Test of the spatial extents of remotely sensed extracted data

**Fig. 2.**
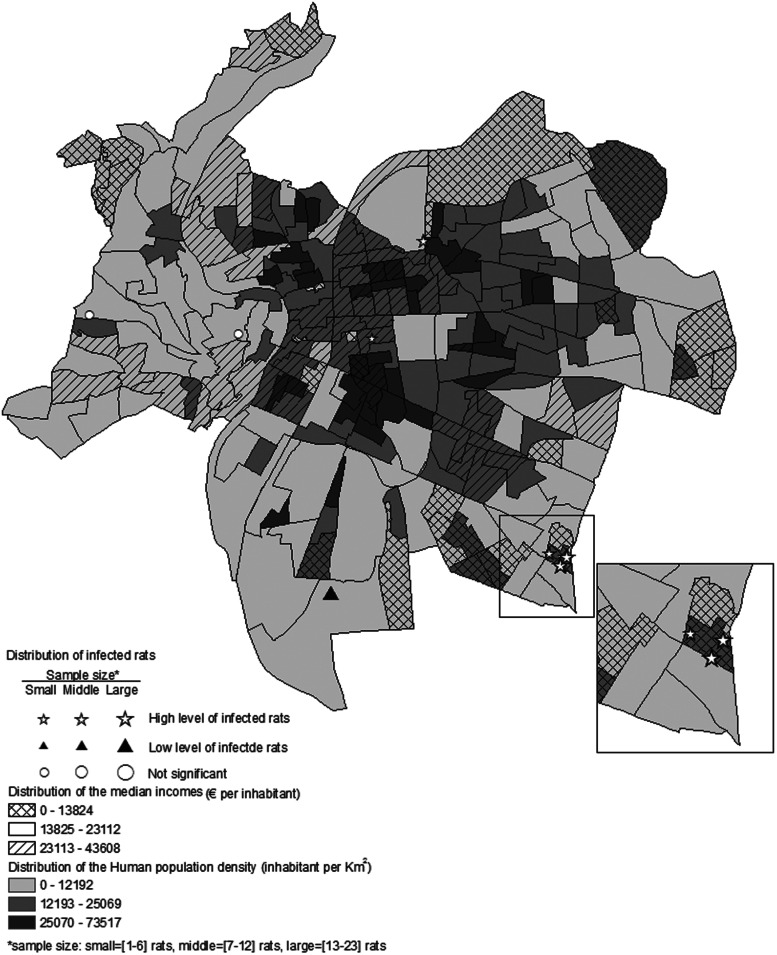
The spatial distribution of human population density and median incomes in Lyon city centre overlaid with high and low prevalence of *L. interrogans* carriage in Norway rats.

The new datasets extracted from the four quadrats were incorporated into the final models selected for *L. interrogans* infection status (see Supplementary online material). The *P* values associated with the relationship between infection status and the covariates decreased with increasing quadrat size. Larger quadrats displayed a better goodness-of-fit model (Table 4). Spatial distributions of the covariates are shown in Figure 2; areas of higher and lower levels of *L. interrogans* infection were mainly located in densely populated and low-income areas.

**Table 4.** Adjusted odds ratios using the final GLM or GLMM and incorporating the human population density (HPD) and median income (MI) by quadrat (a, b, c, d)

Covariates		*L. interrogans* status*			SEOV status†			HEV status‡		
		aOR	95% CI	*P*	aOR	95% CI	*P*	aOR	95% CI	*P*
Maturity	Juveniles	Ref.			Ref.			Ref.		
	Adults	**3·4**	**(1·4–8·5)**	**0·01**	9·3	(0·7–124)	0·09	2·1	(0·4–11·4)	0·38
HPD (a)		**2·0**	**(1·4–2·9)**	**0·01**	—	—	—	—	—	—
MI (a)		0·7	(0·5–1·0)	0·19	0·4	(0·03–5·8)	0·44	0·3	(0·1–0·7)	0·007
Maturity	Juveniles	Ref.			Ref.			Ref.		
	Adults	**3·6**	**(1·8–8·9)**	**0·02**	7·9	(0·6–100)	0·11	3·9	(0·6–24·8)	0·14
HPD (b)		**2·1**	**(1·5–3·0)**	**<0·001**	—	—	—	—	—	—
MI (b)		**0·6**	**(0·4–0·8)**	**0·02**	0·01	(0·0–1·7)	0·08	0·5	(0·1–2·1)	0·38
Maturity	Juveniles	Ref.			Ref.			Ref.		
	Adults	**3·2**	**(1·3–8·3)**	**0·04**	7·9	(0·6–100)	0·11	4·6	(0·8–27)	0·03
HPD (c)		**2·8**	**(2·0–4·1)**	**<0·001**	—	—	—	—	—	—
MI (c)		**0·4**	**(0·3–0·7)**	**0·007**	**0·006**	**(0·0–0·6)**	**0·03**	0·6	(0·1–2·1)	0·41
Maturity	Juveniles	Ref.			Ref.			Ref.		
	Adults	**3·8**	**(1·5–9·3)**	**0·01**	6·8	(0·5–84)	0·13	4·3	(0·7–27·9)	0·126
HPD (d)		**2·3**	**(1·6–3·4)**	**<0·001**	—	—	—	—	—	—
MI (d)		**0·5**	**(0·3–0·8)**	**0·02**	0·02	(0·0–0·4)	0·009	0·7	(0·2–2·6)	0·63

aOR, Adjusted odds ratio; CI, confidence interval.

* aOR, 95% CI and *P* values obtained using the final *L. interrogans* GLM.

† aOR, 95% CI and *P* values obtained using the final SEOV GLMM.

‡ aOR, 95% CI and *P* values obtained using the final HEV GLMM.

## DISCUSSION

This study evaluated the risks of four potentially zoonotic pathogens in Norway rats in Lyon. We demonstrated that rats in human habitats were infected or exposed and probably shed *L. interrogans*, SEOV, HEV, and *T. gondii*. Based on logistic regression models, our study suggests that populated and/or low-income areas of the Rhône department may encompass an increased number of infected rats. Below, the spatially explicit approach and detection methods are expanded upon to explain how the identified risk factors might be used in targeted surveillances.

### Spatial analysis methodology

The risk analysis demonstrated an overlap of densely populated, low-income urbanized areas and Norway rats with *L. interrogans*, SEOV, and HEV infections. Socioeconomic data collected by the INSEE census are most commonly aggregated at the administrative boundary or census areal unit level. Spatial scale can affect the strength and significance of statistical associations, which is known as the modifiable areal unit problem (MAUP) [23]. To minimize the MAUP effect in statistical inference, the covariates can be analysed at hierarchical levels of areal units from the finest to the coarsest resolution to confirm consistent model results [24]. Five hierarchical levels of census units were used in this study. Only the smallest unit failed to confirm the relationship between socioeconomic data and *L. interrogans* positivity, suggesting that HPD and median incomes are robust covariates in predicting the *L. interrogans* infection status in rats, as the ORs were similar when the spatial scale was changed. The ability of HPD and income to predict *L. interrogans* infection in rats aligns with previous studies demonstrating the distribution of human leptospirosis in impoverished inner-city areas [25].

### Pathogen detection

Generally, screening tests used in wildlife species were developed for humans or domestic species and do not have gold standards, as in the case of wild rats. Bacteria recovery from an individual kidney by bacteriological culture was used as the definitive diagnostic of *L. interrogans* and was considered to be appropriate for early detection because kidney colonisation occurs in the first week of *Leptospira* infection [26]. However, this culturing method is insensitive, and the bacterial isolation frequency is typically low. Thus, qPCR was also performed because it is reportedly the most sensitive human analysis [27]. False negatives can still be obtained when PCR is used for undescribed pathogenic strains [28] or the bacteria are aggregated in an unsampled part of the kidney. Therefore, a combination of culture and qPCR was relevant; one positive culture and negative PCR were observed in this study. This combination is expected to provide a reliable reflection of *Leptospira* infection in rats. Nevertheless, we found that negative tests were more likely to occur in dead rats for which PCR inhibitors might be responsible for false-negative results [29]. This result suggests that the prevalence, although consistent with a previous survey in France, might be underestimated [4].

The previously reported sequences of SEOV and HEV [18, 19] demonstrate their presence in individuals. Although several lung and liver lobes were sampled, detection may fail when the viral load is not homogeneously distributed throughout the organs, and areas clear of infection are collected. The combination of HEV-PCR detection on liver and faeces optimized HEV RNA detection; indeed, two individuals were liver-negative and faeces-positive, suggesting that this study may provide an appropriate reflection of rat infection. In addition, the observed prevalence was consistent with previous surveys [30, 31]. Conversely, the prevalence of SEOV might be underestimated.

In domestic animals, the MAT titres of 1:20–1:25 have been validated to detect IgG antibodies to *T. gondii* in sera [32]. The choice of cut-off is usually raised when interpreting serological tests developed for domestic species or humans. In other wildlife species, 1:6 is a biologically relevant threshold because many individuals with titres of 1:6 carry *Toxoplasma* cysts [33]. However, given the low antibody levels of some individuals, all individuals were bioassayed when possible. The absence of *Toxoplasma* detection in mice could be caused by a previous infection that resulted in persistent antibodies, the absence of the parasite in the heart, or parasite death during sample transport. Nonetheless, the 8% of rats exposed to *T. gondii* in the Lyon region and the variation between sites was in agreement with a recent survey in France [34].

### Towards a RBZ-targeted surveillance

Considering the propensity of rats to carry zoonotic pathogens, a public health surveillance programme should be developed. However, using aggregated data may misestimate the prevalence, as aggregate data have a heterogeneous spatial distribution. Thus, risk-based surveillance combining the risk of zoonotic pathogen shedding in rats and the risk of rat-human proximity should be developed to assess and mitigate the spread of RBZs. As the transmission of the four pathogens in rats relies on direct (i.e. behavioural) and indirect (i.e. environmental) contacts, both features should be considered to target surveillance and interpret results.

The zoonosis carriage risk in rats appears to be influenced by the colony's age structure. In our study, sexual maturity (particularly increased age) was identified as a risk factor for *L. interrogans*, SEOV, and HEV, as previously described [35, 36]. The potential influence of age on infection status may result from the presence of maternal antibodies, which can persist for several months in rat neonates [37]. Rat infections are also more likely to be acquired with exploratory behaviour development, which increases with sexual maturity. No male bias was revealed in this study although the likelihood of males acquiring SEOV would be greater due to them having more aggressive encounters and as wounds would be the primary route of SEOV transmission between adult males [38]. Nevertheless, a longitudinal study on rodents revealed that the male bias observed in the prevalence of antibody to hantaviruses varied among trapping sites and was more likely due to habitat, social structure or behavioural variations [39]. The absence of male bias in the present study may be related to its short-term design or to site criteria. Thus, stratified sampling and the colony's age structure should be considered to provide a relevant estimate of RBZ carriage.

We observed a robust relationship between rat infection and socioeconomic covariates which more likely results from a confounding effect of the socio-economic factors with genuine causal factors. The micro-environment features are suspected to be a causal factor involved in pathogen transmission. As Himsworth *et al*. suggested regarding the relationship between micro-environmental features and *Leptospira* prevalence (i.e. environmental factors likely related to variations in *Leptospira* prevalence) [40], our study additionally suggests to focus on environments associated with dense populations and areas with lower incomes. In this way, the results obtained partially answer a previous report on RBZs highlighting the necessity for public health officials to define environmental features that promote pathogen transmission in rats [41]. By suggesting the infected rat distribution patterns, the socio-economic factors could aid targeting and enhancing surveillance. In addition, the effect of socio-economic factors could result from the related effect of rat population size which may increase in lower income areas because of lower hygiene and lower pest control measures. In our study, the effect of the capture success on the number of infected rats was not revealed and could be explained by its low ability to estimate the population size or specific transmission schemes not population-size-related. Indeed, the rat capture success does not merely depend on population size but also on resources, shelter, age structure of the population or pre-poisoning. Furthermore, the usual method of rodent trapping using grids or transects may not be adapted to urban environments since there are a number of physical barriers as well as adverse effects of human activities. The understanding of the potential ways of pathogen transmissions (vertical *vs*. horizontal, direct contact *vs*. environment) and their relative importance is limited. The population density effect on pathogen prevalence might not be the rule in rat colonies as previously suggested for *L. interrogans* infections [40] although it may depends on the pathogens or colonies. As there is no standard to assess the urban rat population size, a critical lack of information limits our study to exploratory work dedicated to suggesting new approaches for the study of RBZ risks.

## CONCLUSION

This study enhances knowledge of the spatial distribution of zoonotic pathogens in Norway rats from the Rhône department. The results suggest that the risk of RBZs is greater in densely populated and lower income areas of Rhône. Given the unprecedented rate of global urbanization and poverty, this study suggests the development of appropriate risk-based surveillance of zoonotic pathogens in Norway rats by targeting reliable indicators of areas with high exposure risks. Although further investigations are required to confirm and refine these findings and to assess the exposure risk in humans, this type of surveillance is required to assess and mitigate the spread of RBZs and to control their potential transmission to humans.

As part of a comprehensive risk assessment, this study should improve prevention measures in the context of a typical ‘One Health’ approach.

## Supplementary material

For supplementary material accompanying this paper visit http://dx.doi.org/10.1017/S0950268814001137. click here to view supplementary material
